# Demonstration of 85% pump depletion and 10^−6^ noise content in quasi-parametric chirped-pulse amplification

**DOI:** 10.1038/s41377-022-00967-6

**Published:** 2022-09-13

**Authors:** Jingui Ma, Kainan Xiong, Peng Yuan, Xiaoniu Tu, Jing Wang, Guoqiang Xie, Yanqing Zheng, Liejia Qian

**Affiliations:** 1 Key Laboratory for Laser Plasmas (MOE), Collaborative Innovation Center of IFSA (CICIFSA), School of Physics and Astronomy, Shanghai Jiao Tong University, Shanghai, 200240 China; 2 Shanghai Institute of Ceramics, Chinese Academy of Sciences, Shanghai, 201800 China; 3 School of Material Science and Chemical Engineering, Ningbo University, Ningbo, Zhejiang 315211 China; 4 Tsung-Dao Lee Institute, Shanghai Jiao Tong University, Shanghai, 200240 China

**Keywords:** High-field lasers, Nonlinear optics, Ultrafast photonics

## Abstract

Full pump depletion corresponds to the upper limit of the generated signal photons relative to the pump pulse; this allows the highest peak power to be produced in a unit area of ultraintense laser amplifiers. In practical systems based on optical parametric chirped-pulse amplification, however, the typical pump depletion is only ~35%. Here, we report quasi-parametric chirped-pulse amplification (QPCPA) with a specially designed 8-cm-thick Sm:YCOB crystal that highly dissipates the idler and hence improves pump depletion. We demonstrate 56% QPCPA energy efficiency for an 810-nm signal converted from a 532-nm pump, or equivalently 85% pump depletion. As another advantage, such a record high depletion greatly suppresses the parametric superfluorescence noise in QPCPA to only ~1.5 × 10^−6^ relative to the amplified signal energy. These results pave the way to beyond the ten-petawatt peak power of the currently most intense lasers.

## Short summary

We demonstrate nearly full pump depletion in quasi-parametric chirped-pulse amplification under the strong saturation regime, enabling a record high signal efficiency of 56% and a low noise content of 10^−6^.

## Graphical abstract

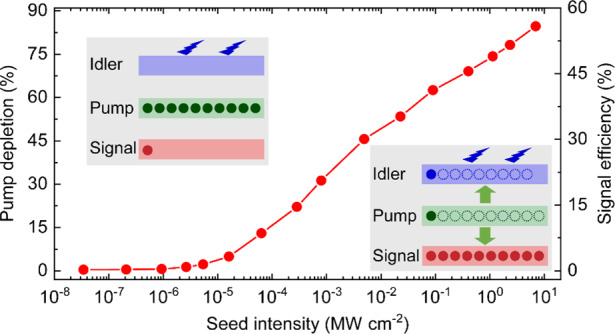

## Introduction

Optical parametric chirped-pulse amplification (OPCPA) has been regarded as the cornerstone of third-generation ultraintense lasers with both ultrahigh peak power and average power^1^. Owing to their broader gain spectrum and adjustable wavelength, OPCPA lasers have generated intense few-cycle pulses from the near-infrared to mid-infrared regions^2–9^; OPCPA laser facilities with peak powers as high as petawatts have also been built in several countries^10–12^. These OPCPA-based ultraintense lasers have promoted advancements in the frontiers of ultrafast science and strong-field physics^13,14^.

Pump depletion is crucial to the overall performance of an OPCPA laser. On the one hand, strong pump depletion means a high conversion efficiency from pump to signal, and allows a high peak power to be produced in a unit area of ultraintense laser amplifiers. In particular, high efficiency is necessary for a petawatt-class OPCPA laser because high pump energy will be limited by optical damage with a given aperture and becomes unreasonably costly. On the other hand, strong pump depletion can suppress the spontaneous parametric superfluoresecence (PSF) in OPCPA, which will improve both the amplified signal pulse stability and energy contrast ratio between the signal and PSF noise^15–19^. Suppression of PSF noise is the precondition to clean the laser-plasma interaction; otherwise, the experimental target will be modified before the arrival of the main pulse once the PSF noise exceeds the ionization threshold.

Unfortunately, achieving strong pump depletion is difficult for OPCPA due to the inherent effect of back conversion^20,21^. Owing to the unmatched photon numbers among the pump, signal, and idler, back conversion of OPCPA, i.e., photon annihilation of the signal and idler, will always occur. When the pump is nonuniform in both space and time, back conversion will occur out of order in spatiotemporal coordinates and is detrimental to OPCPA efficiency. This makes simultaneous achievement of full pump depletion for all spatiotemporal coordinates impossible, thereby limiting the overall pump depletion. For green-laser-pumped OPCPAs at 800 nm, the pump depletion is typically only ~35%^22–26^, as listed in Table 1. Uniform pumping is a straightforward approach to enhance pump depletion, which requires shaping both the pulse and beam into a flattop profile^27,28^. However, temporal shaping is intricate for nanosecond pulses and currently not possible for picosecond pulses.

**Table 1:** Typical pump depletions of reported OPCPA systems

Scheme	*λ*_p_	*λ*_s_	*η*_p_	*η*_s_	Crystal	Reference
OPCPA	532	805	33%	22%	BBO	^22^
	532	850	32%	20%	BBO	^23^
	515	775	36%	24%	BBO	^24^
	515	900	28%	16%	LBO	^25^
	527	790	37%	25%	LBO	^10^
	527	800	15%	9.6%	YCOB	^26^
QPCPA	532	810	85%	56%	Sm:YCOB	This work

λ_*p*_ pump wavelength in nm, λ_*s*_ signal wavelength in nm, η_*p*_, pump depletion, η_*s*_ signal efficiency

Impeding back conversion is a fundamental approach to full pump depletion. In 2015, we proposed the concept of quasi-parametric chirped-pulse amplification (QPCPA), a variant of OPCPA, which can impede back conversion by dissipating the byproduct idler through absorption^29^. Seventy percent pump depletion with a 15-GW peak power was demonstrated in a proof-of-principle experiment with a 3-cm-thick Sm:YCOB crystal oriented in the principal plane. In this work on QPCPA, we demonstrate a pump depletion up to 85% and a peak power up to 0.36 TW with a specially designed 8-cm-thick Sm:YCOB crystal oriented outside the principal plane that has a maximized nonlinear coefficient. Furthermore, we study the PSF characteristic of QPCPA for the first time and observe the significant suppression of PSF noise by strong pump depletion. The measured PSF noise within the signal beam and spectrum is only ~1.5 × 10^‒6^ relative to the amplified signal energy, suggesting a temporally high intensity contrast of ~10^9^ by taking into consideration the signal pulse compression. The QPCPA scheme with strong pump depletion and suppressed PSF noise may adapt current petawatt lasers to higher peak power and will be attractive for a wide range of applications that concern full photon conversion in an ultrafast nonlinear process.

## Results

**Fig. 1:**
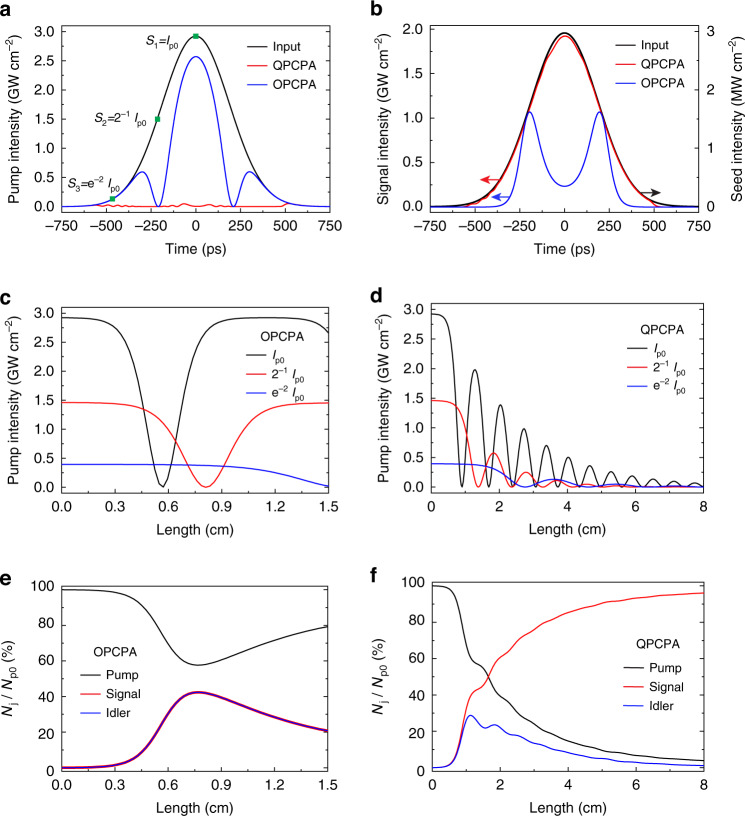
Numerical comparisons of QPCPA and OPCPA. **a** Pump pulses at *L* = 8 cm for QPCPA (red) and *L* = 0.75 cm for OPCPA (blue). The input pump has a peak intensity of *I*_p0_ = 3 GW cm^−2^ (black). **b** Signal pulses at *L* = 8 cm for QPCPA (red) and *L* = 0.75 cm for OPCPA (blue). The input signal has a peak intensity of 3 MW cm^−2^ (black). **c** Evolution of the three OPCPA pump intensities marked in **a**. **d** Evolution of the three QPCPA pump intensities marked in **a**. **e** Evolutions of OPCPA photon numbers *N*_j_ relative to the input pump photon number *N*_p0_, where *j* = *p*, *s*, and *i* represent pump, signal, and idler, respectively. **f** Evolution of the QPCPA photon number. See Methods for simulation parameter details

Numerical simulations are first used to evaluate the ability of QPCPA to achieve strong pump depletion (see Methods for details). A 8-cm-thick Sm:YCOB serves as the QPCPA crystal with idler absorption around 1550 nm. The 532-nm pump and 810-nm signal seed are assumed as Gaussian pulses and beams with peak intensities of 3 GW cm^‒2^ and 3 MW cm^‒2^, respectively, and their pulse durations and beam widths are same to the experimental parameters (Fig. 1a, b). The evolution of three pump-slice intensities (marked by green squares in Fig. 1a) and photon numbers of three interacting waves are traced along the crystal. For comparison, the OPCPA with a 1.5-cm-thick β-BBO crystal is also studied under the same input conditions.

In OPCPA, all three pump slices with different intensities can be fully depleted and then back converted, and they periodically oscillate between their peaks and zero; however, their oscillations are not in order, so they cannot simultaneously reach full depletion at the same crystal length (Fig. 1c). Consequently, the total pump photon number obtained by integrating over all intensities no longer oscillates regularly (Fig. 1e). The pump depletion maximizes to 42% at the crystal length *L* = 0.75 cm, and then gradually decreases to ~20%. The maximum pump depletion of 42% and signal efficiency of 27% are both similar to those of previous experiments listed in Table 1. At the highest pump-depletion, back conversion still occurs around the central peak of the pulse (blue curves in Fig. 1a, b).

QPCPA displays an amplification behavior quite different from OPCPA. Dissipated by idler absorption, the back-conversion effect can be effectively impeded. All the pump slices with different intensities eventually approach full depletion over a long crystal length (Fig. 1d). This allows continuous depletion of total pump photons along the crystal, which can reach 96% at *L* = 8 cm (Fig. 1f). The pump pulse after QPCPA also reflects nearly complete depletion (red curve in Fig. 1a). With the continuous depletion of the pump, the signal energy efficiency continues to increase up to 63%. As a result, the output signal has nearly the same pulse as the input pump (red curve in Fig. 1b). Figure 1 illustrates the great potential of QPCPA to achieve full pump depletion.

**Fig. 2:**
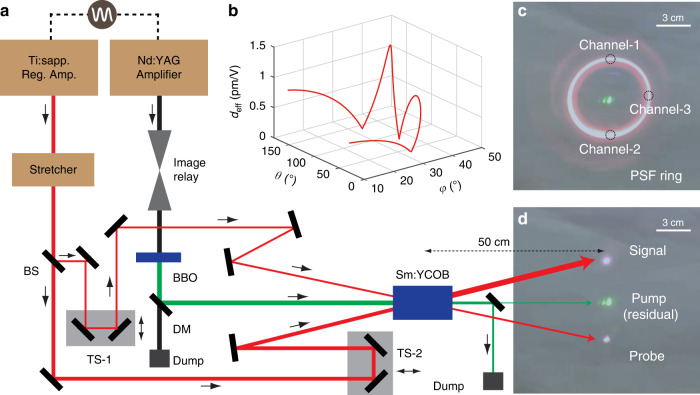
System layout and beam photograph. **a** Single-stage QPCPA setup with a Sm:YCOB crystal and two electrically synchronized lasers. In addition to the pump and signal, a weak probe at 810 nm is also introduced to measure the small-signal gain. BS beam splitter, DM dichroic mirror, TS translation stage. **b** Effective nonlinear coefficient *d*_eff_ of the Sm:YCOB crystal in the phase-matching plane (*θ*, *φ*). **c** PSF ring without a signal seed. Channel 1 for signal amplification; Channel 2 for probing the small-signal gain; Channel 3 for detecting the PSF energy. These three channels are on the same ring of 810-nm wavelength and have an equal aperture of 0.4 cm. **d** Signal and probe beam spots with a strong signal seed

A longer crystal helps enhance pump depletion, as shown in Fig. 1. In the QPCPA experiments with a long crystal, a Sm:YCOB bulk with a size of Φ8.6 cm × 14.3 cm is grown by Czochralski method (see Methods for details), in which the doped rare-earth ions Sm^3+^ can introduce broadband absorption of the idler (black curve in Fig. 5a). The as-grown bulk is then cut into a cuboid crystal with a size of 3.5 × 3.5 × 8.0 cm^3^ along the orientation (*θ* = 114.1°, *φ* = 39.8°). Such an orientation is chosen based on two considerations. On the one hand, the orientation outside the principal plane helps in making a QPCPA crystal as long as possible from the as-grown bulk. The Czochralski growth method of Sm:YCOB favors a longer crystal bulk along the *Y* axis. The phase-matching orientation outside the XZ plane enables the beam propagation direction close to the Y axis, so a longer crystal can be achieved. On the other hand, this orientation supports both magic phase-matching ~810 nm and the maximum effective nonlinear coefficient (Fig. 2b). The calculated nonlinear coefficient is as high as 1.39 pm V^‒1^, nearly two times that in the previously used XZ principal plane (Table 2). A larger nonlinear coefficient can boost pump depletion with a limited crystal length.

**Table 2:** Crystal specifications of Sm:YCOB and YCOB under magic phase matching and a 532-nm pump

Crystal	*λ*_magic_	(*θ*, *φ*)	*d*_eff_	*ρ*_nc_	*ρ*_wo_
Sm:YCOB	810	(114.1°, 39.8°)	1.39	2.70°	0.96°
	816	(15.9°, 180°)	0.67	2.86°	0.95°
YCOB	815	(113.8°, 35.2°)	1.43	3.40°	2.00°
	823	(27.1°, 180°)	0.95	3.56°	1.90°

λ_*magic*_ signal wavelength for magic phase-matching (nm); (*θ*, *φ*), phase-matching orientations relative to the *Z* and *X* crystal axes. *d*_eff_, nonlinear coefficient (pm V^‒1^); *ρ*_nc_, noncollinear angle between the pump and signal; *ρ*_wo_, walk-off angle

**Fig. 3:**
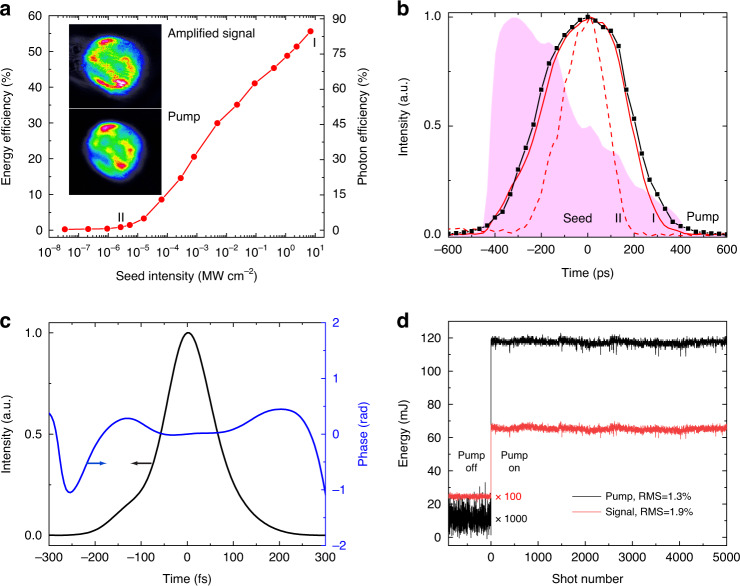
Highly efficient signal amplification. **a** Pump-to-signal efficiency and pump depletion versus seed intensity at a pump intensity of ~3 GW cm^−2^. Inset, beam profiles of the amplified signal and pump. **b** Pulse profiles of the pump (black), amplified signal at seed intensities of 7 MW cm^−2^ (red solid, point I marked in **a**) and 2.5 W cm^−2^ (red dashed, point II marked in **a**). The shaded area shows the chirped-pulse profile (spectrum) of the signal seed. The chirped-pulse of the signal is linearly mapped by its spectrum with a coefficient of 40 ps nm^‒1^. **c** Intensity (black) and phase (blue) of the compressed signal pulse (GRENOUILLE 8-50-USB, Swamp Optics). **d** Five-thousand shots of pump energy (black), and amplified signal energy (red). The measurements are performed by two identical energy meters and begin slightly before the pump turn-on

A single-stage QPCPA system is designed based on the 8-cm-thick Sm:YCOB crystal, which is antireflection coated for both the pump and signal. The experimental setup is schematically plotted in Fig. 2a. The 532-nm pump pulse of 125-mJ energy and 440-ps duration (full width at half maximum, FWHM) is the second harmonic of a 10 Hz Nd:YAG laser (Spit Light, Innolas). The transmission loss of the 80-mm-thick Sm:YCOB crystal is measured to be ~6.5%, so only ~117 mJ pump energy is available for QPCPA. To match the magic phase-matching wavelength of 810 nm and accommodate the crystal absorption spectrum ~1550 nm (black curve in Fig. 5a), the signal pulse from a Ti:sapphire regenerative amplifier (Legend Elite, Coherent) is filtered into a spectral range from 800 to 820 nm (shaded area in Fig. 3b) by an Öffner stretcher and then stretched to cover the pump pulse (black curve in Fig. 3b). After spectral filtering and pulse stretching, the available seed energy for QPCPA is ~0.38 mJ. The pump and signal pulses, synchronized by an electronic phase-locking loop (SynchroLock AP, Coherent), undergo noncollinear interactions with the Sm:YCOB crystal at an external angle of 3.9°. A walk-off-compensated noncollinear configuration is used to minimize the spatial walk-off between the pump and signal. The pump beam with a 3.5 mm diameter has an intensity of ~3 GW cm^‒2^, while the signal beam is telescoped into a 6 mm diameter for full overlap with the pump beam.

The QPCPA saturation is boosted by the seed intensity. With increasing seed intensity, the amplified signal keeps growing without energy back conversion (red curve in Fig. 3a), a characteristic quite similar to that of a conventional laser amplifier such as Ti:sapphire. Notably, the signal efficiency is steeply increasing with the seed on a log-linear scale (Fig. 3a), while it actually manifests the standard saturation characteristic. At the highest seed intensity available in our experiment, the largest output signal energy is ~65 mJ, corresponding to an energy conversion efficiency of ~56% and a photon efficiency (equivalent to pump depletion) of ~85%. At the largest signal output energy, the residual pump energy after QPCPA is ~17.5 mJ, which is the direct indicator of 85% pump depletion. Such a pump depletion level exceeds those of all reported OPCPA systems^22–26^ to the best of our knowledge and even Ti:sapphire laser amplifiers^30^. The signal spectrum (equivalent to the chirped-pulse temporal profile under a mapping coefficient of 40 ps nm^‒1^) becomes stronger and broader with increasing pump depletion (red curves in Fig. 3b). Under the highest pump depletion, both the pulse profile (Fig. 3b) and beam profile (inset in Fig. 3a) of the amplified signal look very similar to those of the pump, which is a result of reaching deep saturation. The signal energy fluctuation under the highest pump depletion is measured to be 1.9% (root mean square, RMS) over 5000 pulse shots (Fig. 3d), which is attributed to the pump fluctuation of 1.3% in RMS. Notably, no thermal effect is observed in the current QPCPA device with crystal absorption. The signal energy instantaneously reaches its steady value once the pump is turned on. The amplified signal pulses can be compressed into an ~120 fs pulse duration by a single-grating compressor^31^ (Fig. 3c). The femtosecond signal has a pulse energy of ~45 mJ after compression, suggesting a peak power of 0.36 TW.

**Fig. 4:**
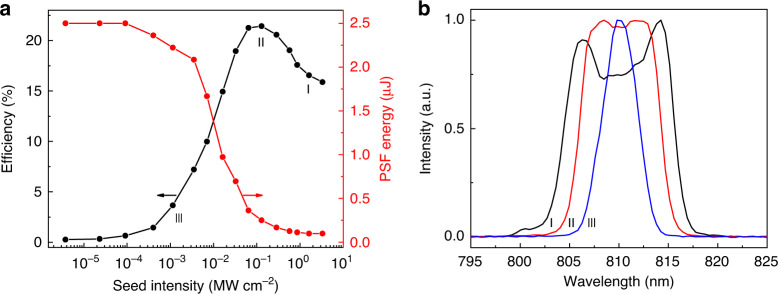
OPCPA results. **a** Evolution of signal efficiency (black) and PSF energy (red). **b** Amplified signal spectra at seed intensities of 1.6 MW cm^−2^ (black curve, point I marked in **a**), 130 kW cm^−2^ (red curve, point II marked in **a**), and 1 kW cm^−2^ (blue curve, point III marked in **a**), respectively

An OPCPA experiment is carried out for the comparison with QPCPA (Fig. 4). A 1.5-cm-thick *β*-BBO crystal is adopted in OPCPA and other experimental parameters remain the same. In contrast to QPCPA, the OPCPA efficiency initially increases with the seed and then degrades after the maximum value (black curve in Fig. 4a), which is the signature of the back-conversion effect. The highest OPCPA efficiency of 21.5% is much lower than the QPCPA efficiency of 56%. The evolution of the amplified signal spectrum in OPCPA is also different from that in QPCPA. The OPCPA spectrum gets broad in the beginning, then becomes flat, and finally shows a central dip caused by the back conversion effect (Fig. 4b). Clearly, there is a trade-off between the conversion efficiency and spectral bandwidth in OPCPA. By contrast, the spectral bandwidth in QPCPA always increases with efficiency.

**Fig. 5:**
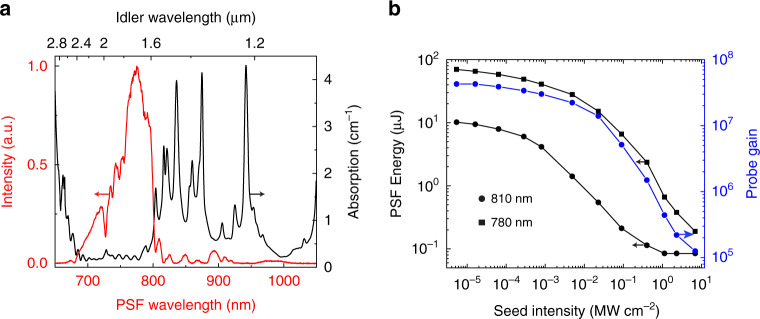
Suppression of PSF noise by QPCPA. **a** Measured full PSF spectrum (red) and idler absorption spectrum of the Sm:YCOB crystal (black). **b** Evolution of the PSF energy (black squares and circles) and probed small-signal gain (blue circles). As shown in Fig. 2c, the PSF energies within a 0.4-cm aperture are detected after two 10-nm bandpass filters of 810 nm (circles) and 780 nm (squares)

The PSF characteristic of QPCPA is studied experimentally. The high small-signal gain contributed by both the strong pump and long crystal facilitates observation and study of PSF noise in our experiment. Owing to the noncollinear phase-matching configuration, the pump pulses excite observable PSF with a clear ring structure when the signal seed is blocked, as illustrated in Fig. 2c. The angular diameter of the brightest PSF ring is ~136 mrad. The full PSF spectrum (red curve in Fig. 5a) exhibits a significant dependence on the idler absorption spectrum (black curve in Fig. 5a). The majority of PSF energy lies in the wavelength range between 700 and 800 nm, where the corresponding idler absorption is negligible. This part of the PSF spectrum dominates the bright ring. In addition, there are some distinguishable structures inside (long wavelength) and outside (short wavelength) the bright PSF ring (Fig. 2c), which are very weak due to the strong idler absorption.

Once the signal is seeded, the PSF noise is significantly suppressed (Fig. 2d). Although the signal occupies only a small portion of the cone (Channel 1), high pump depletion induced by efficient signal amplification can suppress the whole PSF ring. This provides a convenient method to measure the PSF content within the amplified signal beam. We can monitor the PSF energy at Channel 3 (Fig. 2c) on the PSF ring that is away from the signal channel (Channel 1 in Fig. 2c). Similarly, another channel (Channel 2 in Fig. 2c) is used to probe the small-signal gain by seeding ultraweak light. The probe light, split from the signal seed, is gradually attenuated with neutral density filters until the amplified probe light can just be distinguished from the PSF background. This can avoid the effect of probe-induced pump depletion on the measurement of the small-signal gain. All three channels are on the same ring of 810-nm wavelength and are restricted to the same size of 4 mm (the beam size of the amplified signal) with three apertures. The amplified signal from Channel 1 is measured by an energy meter, while the amplified probe light from Channel 2 and PSF from Channel 3 are measured by two identical photodiodes (DET10A/M, Thorlabs). The amplified probe light from Channel 2 is high enough to be recorded by an energy meter when there is no signal seed, which is used to relate the photodiode voltage to the absolute energy. A bandpass filter (FBH810-10, Thorlabs) is used in Channel 3 to filter out the PSF noise outside the signal bandwidth (black curve with circles in Fig. 5b); for comparison, we also monitor the PSF noise at ~780 nm with another filter (FBH780-10, Thorlabs), as shown by the black curve with squares in Fig. 5b. The filter transmittance is considered in the data processing. Such an arrangement allows us to monitor the evolution of the small-signal gain and PSF energy in parallel to signal amplification.

Figure 5b summarizes the results. Without a signal seed, the measured PSF energy within the signal bandwidth is only 10 μJ, which is 7 times lower than that at ~780 nm. With increasing signal seed, both PSF noise components at 780 nm and 810 nm are significantly suppressed. At the highest seed intensity, the PSF energy within the signal bandwidth is ~0.1 μJ, with a ratio of 1.5 × 10^‒6^ relative to the amplified signal energy of 65 mJ. Compared to the case of no seed, the largest seed can suppress the PSF noise by more than two orders of magnitude. This can be understood from the measured probe gain (blue curve with circles in Fig. 5b). The probe gain decreases from 4 × 10^7^ to 10^5^ with increasing seed intensity, a result of pump depletion induced by efficient signal amplification. The final 10^5^ probe gain is below the ~10^6^ gain threshold for significant PSF generation^32^, so the PSF noise becomes insignificant at the highest pump depletion. The PSF energy in OPCPA (red curve in Fig. 4a) is also measured. The PSF energy within the area and bandwidth same to the signal is ~2.5 μJ when there is no seed. With the increase of seed intensity, the PSF energy can be reduced. Under the maximum efficiency at the seed intensity of 130 kW cm^−2^, the PSF energy in OPCPA is ~0.25 μJ, which is 10^‒5^ relative to the amplified signal energy of 25 mJ. Based on the comparison of Figs. 4a and 5b, we conclude that the QPCPA can suppress the PSF noise more effectively owing to its stronger pump depletion.

## Discussion

Finally, we briefly analyze the peak-power scalability of QPCPA pumped by a large-energy Nd:glass laser. Under a pump intensity of 3 GW cm^‒2^ and a pulse duration of 3 ns, typically, a 9 J pump energy, below the damage threshold, is allowed in a unit area of a Sm:YCOB crystal (1 × 1 cm^2^). Since Sm:YCOB crystals can potentially reach a large-size of 12 × 12 cm^2^^33^, a maximum energy of ~1300 J can be used as a pump, which can be converted into an ~728 J signal energy under a conversion efficiency of 56%. The signal energy of ~500 J can be expected after compression under a transmission efficiency of 70%. In this work, a compressed pulse duration of ~100 fs is achieved for a spectral range from 800 to 820 nm. According to Fig. 5a, the available QPCPA spectral range with strong idler absorption exceeds 200 nm (800‒1000 nm), so the compressed pulse duration could be as short as ~10 fs. Such an ultrashort pulse duration is reasonably expected based on the ultrabroadband magic phase-matching (the PSF spectrum in Fig. 5a suggests a sufficiently large gain bandwidth based on the current phase-matching configuration) and the robustness of QPCPA against phase mismatch^34,35^. Therefore, the QPCPA scheme based on large-size Sm:YCOB crystals can support a peak power as high as 50 PW by using a pump energy comparable to that of current petawatt laser facilities.

Besides high efficiency and broad bandwidth, the QPCPA device is insensitive to phase mismatch^29,34^, so it can withstand severe thermal dephasing caused by the idler absorption and can allow the signal output with a high average power like OPCPA devices^35^. Actually, no thermal effect is observed in our experimental with a pump power of 1.25 W. As shown in Fig. 3d, the amplified signal energy does not decay with the accumulation of laser shots. The thermal effect might also be negligible in future petawatt lasers with QPCPA, because the high-energy Nd:glass pump lasers have a low repetition rate around one pulse per minute and the deposited absorption power is less than the QPCPA acceptance of ~100 W ^35^.

In conclusion, we have demonstrated a record high pump depletion of 85% and a pump-to-signal efficiency of 56% in a TW-class QPCPA. The PSF noise of QPCPA has been studied experimentally for the first time, and the PSF energy relative to the amplified signal can be as low as 1.5 × 10^‒6^. The demonstrated QPCPA pump depletion is ~2.5 times that of OPCPA, so QPCPA may be a qualified candidate for pushing ultraintense lasers beyond the current 10-PW limit.

## Materials and methods

### Numerical simulation

In the numerical simulation, a three-dimensional (*x*, *y*, *t*) code solves the nonlinear coupled-wave equations including the terms of dispersion, diffraction, and idler absorption^35^. Under the conditions of noncollinear phase-matching and large beam widths used in the experiment, both the temporal and spatial walk-offs among the three interacting waves are negligible, and are thus not taken into account in the simulation. The QPCPA crystal is a 8-cm-thick Sm:YCOB that has an idler absorption of ~1.5 cm^−1^ (black curve in Fig. 5a), while the OPCPA crystal is a 1.5-cm-thick *β*-BBO without absorption. The QPCPA and OPCPA simulations adopt the same input conditions from the experiment (see Experiment details).

### Fabrication of Sm:YCOB

Sm_2_O_3_, Y_2_O_3_, CaCO_3_, and H_3_BO_3_ powders (4 N purity) are used as starting materials. To guarantee batch accuracy, CaCO_3_ and Re_2_O_3_ (Sm_2_O_3_ and Y_2_O_3_) powders are baked at 250 °C and 1200 °C for 10 hours to remove absorbed water, respectively. Polycrystalline materials are prepared by mixing powders in stoichiometric proportions at 1200 °C for 20 hours. A YCOB crystal bar with <010> direction is used as seed, and a Sm_0.3_Y_0.7_COB single crystal is grown using a radio frequency (RF)-heating Czochralski furnace with an iridium crucible. A mixture of N_2_ and 1 vol% O_2_ gas is used for the growth atmosphere. The crystal is pulled at a rate of 0.6-1 mm h^‒1^ with a rotation of 18-22 rpm. After growth, the as-grown crystal is cooled to room temperature within 72 hours to minimize the excess stress exerted on the crystal.

The as-grown crystal is processed according to the phase-matching design. The magic phase matching with a gain bandwidth mainly limited by third-order dispersion is calculated with a 532-nm pump and the Sellmeier equation given in ref. ^34^. The nonlinear coefficient *d*_eff_ is also considered in the calculation process to find a magic phase-matching solution with the largest *d*_eff_. For this purpose, we extend the calculation from the principal plane to all spatial orientations. The calculation is very complex because there are four degrees of magnitude to be determined, i.e., *θ*, *φ*, *ρ*_nc_, and *λ*_s_ (see Table 2 for their definitions). An approximate method is used to simplify the calculation. First, the (*θ*, *φ*) orientation with the largest *d*_eff_ is calculated at a fixed *λ*_s_ in a collinear configuration following the procedure in ref. ^36^. Second, a noncollinear angle *ρ*_nc_ is introduced and adjusted along the above (*θ*, *φ*) orientation to evaluate if the first-order and second-order dispersion terms in the Taylor expansion of the phase mismatch can simultaneously vanish^37^. If not, the first procedure is repeated by choosing a new *λ*_s_. The calculation results are listed in Table 2, where the results for the YCOB crystal are also presented for comparison. The orientation (*θ* = 114.1°, *φ* = 39.8°) is chosen for cutting the Sm:YCOB crystal because it has a large nonlinear coefficient, a moderate noncollinear angle, and a small walk-off angle, more importantly, can allow fabrication of a crystal as long as possible from the as-grown bulk.

### Experiment details

The pump laser source consists of a 1 kHz Nd:YVO_4_ regenerative amplifier (Pico-Regen, HighQ) and a 10 Hz Nd:YAG boost amplifier (Spit Light, Innolas). The 1064 nm laser is image relayed onto a 5-mm-thick β-BBO crystal for frequency doubling. The diffraction-caused aberrations in the 1064-nm beam are filtered out by a far-field pinhole located in a vacuum tube. The generated 532-nm light is shaped into a beam size of 3.5 mm in diameter by a lens assembly before pumping the QPCPA. The temporal profile of the 532-nm pump pulse (black curve in Fig. 3b) is measured by a home-built time-scanning cross-correlator, in which the 35-fs pulse from a Ti:sapphire regenerative amplifier (Legend Elite, Coherent) is used to induce sum-frequency generation with the pump pulse in a 1-mm-thick *β*-BBO crystal.

The Ti:sapphire regenerative amplifier also serves as the seeder of the QPCPA. Its output is shaped in spectrum and stretched in time by an Öffner stretcher. Only the spectral portion from 800 to 820 nm (shadow in Fig. 3b) serves as the QPCPA seed for accommodating the idler absorption spectrum (Fig. 5a), which is achieved by placing an aperture in the middle of the pulse stretcher. The stretcher includes a Au-coated diffraction grating with a line density of 1480 mm^‒1^, a Au-coated concave mirror with a focal length of 1 m and a Au-coated convex mirror with a focal length of 0.5 m, where the distance between the grating and the convex mirror is ~23 cm. The incident angle and diffraction angle on the grating are 22° and 55.5°, respectively. These arrangements enable a dispersion coefficient of ~40 ps nm^‒1^. A small portion of the stretched signal (~20 μJ) is split from the main beam to be used as probe light for measuring the small-signal gain of the QPCPA, and the remaining majority of the signal is used to seed the QPCPA. The signal seed and probe light are fed into the Sm:YCOB crystal with the same noncollinear angle relative to the pump beam, and they lie on the two ends of one diameter of the PSF rings (Fig. 2d). Some neutral density filters are used to control the intensities of the signal seed and probe light. The time delay introduced by these added optical thicknesses is dynamically compensated by the translation stages (TS-1 and TS-2 in Fig. 2a).

## References

[1] Fattahi H (2014). Third-generation femtosecond technology. Optica.

[2] Budriūnas R (2017). 53 W average power CEP-stabilized OPCPA system delivering 5.5 TW few cycle pulses at 1 kHz repetition rate. Opt. Express.

[3] Gu X (2009). Generation of carrier-envelope-phase-stable 2-cycle 740-μJ pulses at 2.1-μm carrier wavelength. Opt. Express.

[4] Windeler MKR (2019). 100 W high-repetition-rate near-infrared optical parametric chirped pulse amplifier. Opt. Lett..

[5] Elu U (2017). High average power and single-cycle pulses from a mid-IR optical parametric chirped pulse amplifier. Optica.

[6] Wang PF (2018). 2.6 mJ/100 Hz CEP-stable near-single-cycle 4 μm laser based on OPCPA and hollow-core fiber compression. Opt. Lett..

[7] von Grafenstein L (2020). Multi-millijoule, few-cycle 5 μm OPCPA at 1 kHz repetition rate. Opt. Lett..

[8] Sanchez D (2016). 7 μm, ultrafast, sub-millijoule-level mid-infrared optical parametric chirped pulse amplifier pumped at 2 μm. Optica.

[9] Qu SZ (2019). 9 μm few-cycle optical parametric chirped-pulse amplifier based on LiGaS_2_. Opt. Lett..

[10] Zeng XM (2017). Multi-petawatt laser facility fully based on optical parametric chirped-pulse amplification. Opt. Lett..

[11] Lozhkarev VV (2007). Compact 0.56 Petawatt laser system based on optical parametric chirped pulse amplification in KD*P crystals. Laser Phys. Lett..

[12] Bromage J (2021). MTW-OPAL: a technology development platform for ultra-intense optical parametric chirped-pulse amplification systems. High. Power Laser Sci. Eng..

[13] Popmintchev T (2012). Bright coherent ultrahigh harmonics in the keV X-ray regime from mid-infrared femtosecond lasers. Science.

[14] Koç A (2021). Compact high-flux hard X-ray source driven by femtosecond mid-infrared pulses at a 1 kHz repetition rate. Opt. Lett..

[15] Manzoni C (2011). Excess quantum noise in optical parametric chirped-pulse amplification. Opt. Express.

[16] Tavella F, Marcinkevičius A, Krausz F (2006). Investigation of the superfluorescence and signal amplification in an ultrabroadband multiterawatt optical parametric chirped pulse amplifier system. N. J. Phys..

[17] Moses J (2009). Highly stable ultrabroadband mid-IR optical parametric chirped-pulse amplifier optimized for superfluorescence suppression. Opt. Lett..

[18] Mikhailova JM (2011). Ultra-high-contrast few-cycle pulses for multipetawatt-class laser technology. Opt. Lett..

[19] Papadopoulos DN (2017). High-contrast 10 fs OPCPA-based front end for multi-PW laser chains. Opt. Lett..

[20] Moses J (2009). Temporal optimization of ultrabroadband high-energy OPCPA. Opt. Express.

[21] Ma JG (2017). Origin and suppression of back conversion in a phase-matched nonlinear frequency down-conversion process. Chin. Opt. Lett..

[22] Herrmann D (2009). Generation of sub-three-cycle, 16 TW light pulses by using noncollinear optical parametric chirped-pulse amplification. Opt. Lett..

[23] Witte S (2006). A source of 2 terawatt, 2.7 cycle laser pulses based on noncollinear optical parametric chirped pulse amplification. Opt. Express.

[24] Mecseki K (2019). High average power 88 W OPCPA system for high-repetition-rate experiments at the LCLS x-ray free-electron laser. Opt. Lett..

[25] Kessel A (2018). Relativistic few-cycle pulses with high contrast from picosecond-pumped OPCPA. Optica.

[26] Yu LH (2012). Experimental demonstration of joule-level non-collinear optical parametric chirped-pulse amplification in yttrium calcium oxyborate. Opt. Lett..

[27] Waxer LJ (2003). High-conversion-efficiency optical parametric chirped-pulse amplification system using spatiotemporally shaped pump pulses. Opt. Lett..

[28] Bagnoud V (2005). 5 Hz, >250 mJ optical parametric chirped-pulse amplifier at 1053 nm. Opt. Lett..

[29] Ma JG (2015). Quasi-parametric amplification of chirped pulses based on a Sm^3+^-doped yttrium calcium oxyborate crystal. Optica.

[30] Chu YX (2015). High-energy large-aperture Ti:sapphire amplifier for 5 PW laser pulses. Opt. Lett..

[31] Lai M, Lai ST, Swinger C (1994). Single-grating laser pulse stretcher and compressor. Appl. Opt..

[32] Acco S, Blau P, Arie A (2008). Output power and spectrum of optical parametric generator in the superfluorescent regime. Opt. Lett..

[33] Tu XN (2018). Research on growth and defects of 5 in. YCOB single crystal. J. Cryst. Growth.

[34] Ma JG (2017). Broadband, efficient, and robust quasi-parametric chirped-pulse amplification. Opt. Express.

[35] Yin Z (2019). Quasi-parametric chirped-pulse amplification simultaneously enables high peak power and high average power. IEEE Photonics J..

[36] Chen CT (2000). Determination of the nonlinear optical coefficients of YCa_4_O(BO_3_)_3_ crystal. J. Optical Soc. Am. B.

[37] Guo XY (2014). Bandwidth analysis of type-I optical parametric chirped pulse amplification systems. J. Optical Soc. Am. B.

